# Glycated Albumin Levels in Patients with Type 2 Diabetes Increase Relative to HbA_1c_ with Time

**DOI:** 10.1155/2015/576306

**Published:** 2015-09-21

**Authors:** Hye-jin Yoon, Yong-ho Lee, Kwang Joon Kim, So Ra Kim, Eun Seok Kang, Bong-Soo Cha, Hyun Chul Lee, Byung-Wan Lee

**Affiliations:** ^1^Division of Endocrinology and Metabolism, Department of Internal Medicine, Yonsei University College of Medicine, Seoul 120-752, Republic of Korea; ^2^Severance Hospital, Seoul, Republic of Korea; ^3^Severance Executive Healthcare Clinic, Yonsei University Health System, Seoul, Republic of Korea

## Abstract

We recently reported that glycated albumin (GA) is increased in subjects with longer duration of diabetes and with decreased insulin secretory function. Based on this, we investigated whether GA increases with time relative to glycated hemoglobin (HbA_1c_) and the association between GA and beta-cell function. We analyzed 340 type 2 diabetes patients whose serum GA and HbA1c levels had been repeatedly measured over 4 years. We assessed the pattern of changes with time in glycemic indices (GA, HbA_1c_, and GA/HbA_1c_ ratio) and their relationship with beta-cell function. In all patients, glycemic indices decreased and maintained low levels around 15 and 27 months. However, from 39 months to 51 months, GA significantly increased but HbA_1c_ tended to increase without statistical significance. We defined ΔGA/HbA_1c_ as the difference between the nadir point (at 15 to 27 months) and the end point (at 39 to 51 months) and found that ΔGA/HbA_1c_ was positively correlated with diabetes duration and negatively related to beta-cell function. In multivariable linear regression analyses, ΔGA/HbA_1c_ was independently associated with diabetes duration. In conclusion, this study demonstrated that serum GA levels increase relative to HbA_1c_ levels with time.

## 1. Introduction

Glucose monitoring is essential for the appropriate care and treatment of patients with diabetes in order to avoid diabetic complications and hypoglycemia. An accurate measure of glucose level allows physicians and patients to make optimal decisions about food, physical activity, and medications [1]. Of the glycemic indices, the American Diabetes Association recommends glycated hemoglobin (HbA_1c_) testing in all diabetic patients as an initial assessment and then as a part of continuing care [2]. This recommendation is derived from clinical data that shows that HbA_1c_ reflects average glycemic status over 2-3 months and predicts diabetic complications [3, 4]. Although HbA_1c_ provides useful information, it might be inadequate in clinical situations such as anemia, renal insufficiency, and gestational diabetes. Glycated albumin (GA) has been gaining popularity as an indicator in several physiologic and pathologic conditions [5] because it provides more information than the gold standard HbA_1c_. In line with this trend, we have demonstrated the clinical relevance of GA in type 2 diabetes mellitus (T2D) with insulin secretory dysfunction rather than insulin resistance [6], fluctuating or poorly controlled glycemic excursions [7], and progressing atherosclerosis [8].

In the natural course of T2D, however, beta-cell function decreases as duration of diabetes increases [9]. Moreover, glycemic excursions worsen due to decreased beta-cell function [10]. In a recent cross-sectional study, we reported that the levels of GA/HbA_1c_ were significantly elevated in subjects with long diabetic duration, largely attributed to the inverse relationships between GA and pancreatic beta-cell secretory indices [11], and suggested that clinicians should be careful in interpreting GA as only an indicator of glycemic control in T2D cases of longer duration. However, no longitudinal studies investigating the change in GA and HbA_1c_ over time in patients with T2D have been published.

In this longitudinal observational study, we investigated the changing pattern of glycemic indices such as GA, HbA_1c_, and GA/HbA_1c_ over 4 years in order to determine whether GA increases more with time relative to HbA_1c_ in subjects with T2D. We also investigated which clinical and biochemical parameters are associated with changes in the GA/HbA_1c_ ratio.

## 2. Research Design and Methods

### 2.1. Subjects and Data Collection

In this longitudinal observational study, we recruited patients with T2D who had enrolled in previous studies [6, 7] between May 2009 and June 2011 and who were followed up in June 2014. Using electronic medical records, we reviewed and rechecked demographic and clinical data for age, gender, metabolic parameters, and duration of diabetes. The diabetic duration was defined from the date the patients were first diagnosed with diabetes by blood tests or by patient recall from interviews.

To investigate the changes in glycemic indices with time, we tried to include patients whose duration of diabetes was less than 5 years. Patients were included if they were (1) aged ≥20 years, (2) had repeated laboratory data for both HbA_1c_ and GA up to the final follow-up point, and (3) had undergone a baseline standardized liquid meal test (Ensure, Meiji Dairies Corporation, Tokyo, Japan; 500 kcal, 17.5 g fat (31.5%), 68.5 g carbohydrate (54.5%), and 17.5 g protein (14.0%)) after an overnight fast. Patients were excluded if they had any medical conditions that could alter HbA_1c_ or GA levels such as liver cirrhosis or chronic kidney diseases (estimated glomerular filtration rate (GFR) by chronic kidney disease epidemiology collaboration formula <60 mL/min/1.73 m^2^), pregnancy, or hematologic disorders or if they were being treated with steroids.

The protocol of this study was approved by the Institutional Review Board at Severance Hospital (IRB numbers 4-2009-0656, 4-2012-0398, and 4-2014-0507). Written informed consent for this study was not required by the Institutional Review Board because researchers only accessed the database for analysis purposes, and personal information was not used.

### 2.2. Laboratory Measurements

The baseline glycemic indices (GA, HbA_1c_, and GA/HbA_1c_) were defined as the values measured at enrollment. Subsequently, serum GA and HbA_1c_ were measured every 3 or 6 months. The end point glycemic indices of each subject were measured between 39 and 51 months. For glucose and C-peptide analyses, blood samples were collected at 0 and 90 min (basal and stimulated values) as part of the standardized liquid meal test. Pancreatic beta-cell functions in the context of ambient insulin secretory function were assessed using the following indices: (1) PCGR (stimulated C-peptide level/stimulated glucose level × 100), (2) C-peptide increment (ΔC-peptide = stimulated C-peptide − basal C-peptide), and (3) C-peptide-genic index [CGI = (stimulated C-peptide − basal C-peptide)/(stimulated glucose − basal glucose)]. Measurement techniques included the hexokinase method for glucose and high-performance liquid chromatography using Variant II Turbo (Bio-Rad Laboratories, Hercules, CA) for HbA_1c_. Serum GA was analyzed by an enzymatic method using an albumin-specific proteinase, ketoamine oxidase, an albumin assay reagent (LUCICA GA-L; Asahi Kasei Pharma Co., Tokyo, Japan), and a Hitachi 7699 P module autoanalyzer (Hitachi Instruments Service, Tokyo, Japan). GA values were calculated from the ratio of GA to total serum albumin and expressed as a percentage. Serum C-peptide levels were measured in duplicate using an immunoradiometric assay method (Beckman Coulter, Fullerton, CA).

### 2.3. Statistical Analysis

All continuous variables were presented as mean ± standard deviation (SD) or median (quartiles) or as mean ± standard error (SE) for variables on the graphs. Categorical variables were described as* N* (%). Differences were analyzed using Student's* t*-test for the continuous variables and the chi-square test for categorical variables.

Repeated measured analysis of variance (ANOVA) with Bonferroni correction and paired* t*-test were used to determine the significance of differences in glycemic indices according to duration of diabetes. We compared GA, HbA_1c_, and GA/HbA_1c_ levels at baseline and 3, 15, 27, and 39 to 51 months after enrollment in all patients who had glycemic values available at that time point. Because all glycemic indices reached their lowest level between 15 and 27 months (arbitrarily defined nadir point), we defined ΔGA/HbA_1c_ as the difference in GA/HbA_1c_ between the end point (39 to 51 months) and nadir point (15 to 27 months). One-way ANOVA with Tukey correction was used to compare the differences of duration of diabetes and PCGR according to the tertiles of ΔGA/HbA_1c_ ratio. Multivariable linear regression analysis was performed to determine the independent relationship of the studied variables including duration of diabetes associated with ΔGA/HbA_1c_ increase. Statistical analyses were performed using PASW Statistics version 18.0 for Windows (SPSS Inc., Chicago, IL, USA).

## 3. Results

### 3.1. Study Population Characteristics

A total of 340 subjects (71% men, mean age 61.3 ± 11.6 years) were enrolled in this study. The patient characteristics of the cohort are shown in Table 1. The mean body mass index (BMI) was 25.4 ± 3.6 kg/m^2^ and the prevalence of hypertension was 57% (*n* = 195). Median duration of diabetes and levels of mean HbA_1c_ were 1 (range: 0–5.0) year and 7.0% + 0.9%, respectively. We also measured baseline insulin secretory beta-cell function indices such as PCGR (3.24 ± 2.1), ΔC-peptide level (4.13 ± 2.6), and CGI (0.08 ± 0.4). All glycemic indices, GA (19.3 ± 6.6 versus 16.5 ± 4.9), HbA_1c_ (7.7 ± 1.6 versus 7.0 ± 1.2), and GA/HbA_1c_ (2.47 ± 0.5 versus 2.33 ± 0.4), were decreased at final follow-up compared to those at baseline. At the time of enrollment, the patients were being treated with metformin (221 patients; 65% of the study population), sulfonylurea (88; 26%), DPP-IV inhibitors (59; 17%), or insulin (63; 19%).

### 3.2. Glycated Albumin and GA/HbA_1c_ Ratio Levels Increased Relative to HbA_1c_ Levels over Time

**Figure 1:**
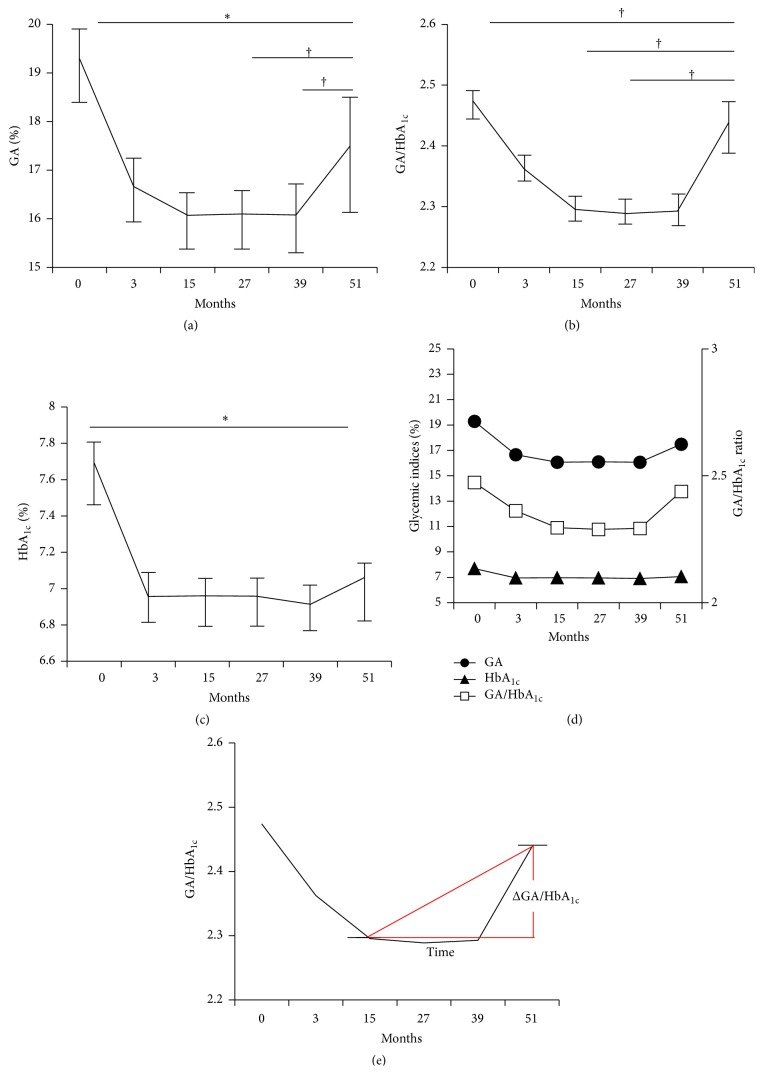
Changing patterns of glycemic indices over 4 years. (a) GA, (b) GA/HbA_1c_ ratio, (c) HbA_1c_, (d) changing patterns of glycemic indices, (e) ΔGA/HbA_1c_, calculated by end point GA/HbA_1c_ – nadir point GA/HbA_1c_. Data are presented as mean with SE. ^*∗*^ *p* < 0.001, ^†^ *p* < 0.05 for the comparison with 51 months.

In all patients, both levels of GA (16.1% ± 4.0%) and GA/HbA_1c_ ratio (2.30 ± 0.4) improved and reached the nadir points on glucose control at 15 months (Figure 1). From 15 months to 39 months, the nadir glycemic indices were stably maintained. From 39 months to 51 months, GA significantly increased (16.1% ± 4.8% to 17.5% ± 4.9%, *p* = 0.028), but HbA_1c_ tended to increase without statistical significance (6.9% ± 1.0% to 7.1% ± 1.2%, *p* = 0.389). The levels of GA at 27 and 39 months (*p* = 0.029 and 0.028, resp.) as well as GA/HbA_1c_ ratio at 15 and 27 months (*p* = 0.038, *p* = 0.039) were significantly lower than at 51 months (Figures 1(a) and 1(b)). However, statistical differences in HbA_1c_ between each time point (3, 15, 27, and 39 months) and the last time point (51 months) were not significant except for baseline (Figure 1(c)). In sum, GA levels and the GA/HbA_1c_ ratio, but not HbA_1c_ levels, were significantly increased at the final follow-up compared with those at the nadir time point (Figure 1(d)).

### 3.3. Associations between ΔGA/HbA_1c_ and Clinical and Biochemical Parameters

Since the GA/HbA_1c_ ratio significantly increased from the nadir point to the final follow-up point, which was designated as ΔGA/HbA_1c_ (Figure 1(e)), we tried to determine the clinical and biochemical parameters that are associated with ΔGA/HbA_1c_ (Table 2). In the univariate linear regression analysis, duration of diabetes (standardized *β* coefficient (STD *β*) = 0.187, *p* = 0.001), mean GA (STD *β* = 0.345, *p* < 0.001), and mean HbA_1c_ (STD *β* = 0.128, *p* = 0.018) were positively associated with ΔGA/HbA_1c_. On the other hand, beta-cell function indices were negatively related to ΔGA/HbA_1c_. In particular, PCGR (STD *β* = −0.145, *p* = 0.007) was more strongly associated with ΔGA/HbA_1c_ than ΔC-peptide (STD *β* = −0.139, *p* = 0.011).

**Figure 2:**
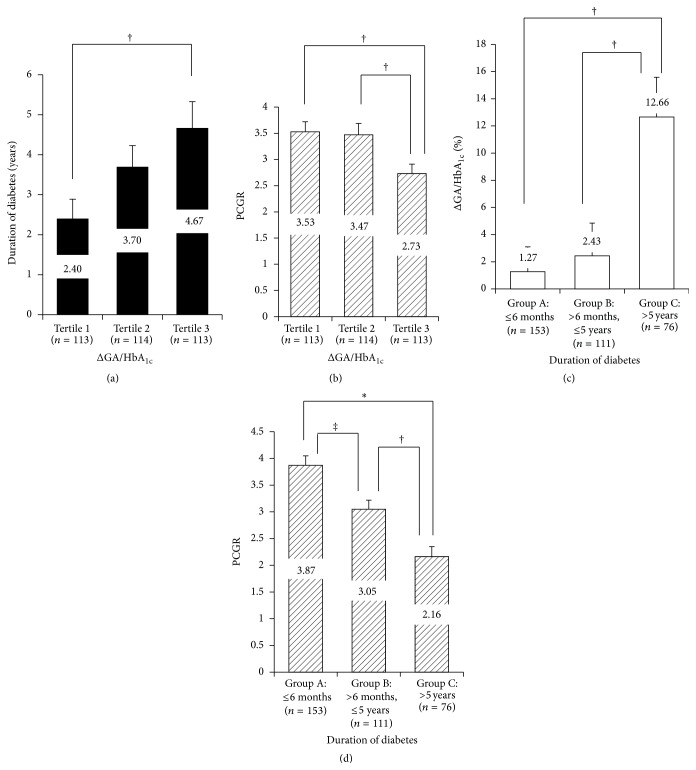
Correlations between ΔGA/HbA_1c_ and duration of diabetes, beta-cell function. (a, b) Differences of duration of diabetes (a) and PCGR (b) in subjects according to the tertiles of ΔGA/HbA_1c_. (c, d) Differences of ΔGA/HbA_1c_ (c) and PCGR (d) in subjects according to duration of diabetes. ^†^ *p* < 0.05, ^‡^ *p* < 0.01, ^*∗*^ *p* < 0.001; ΔGA/HbA_1c_ (%) = ΔGA/HbA_1c_/nadir point GA/HbA_1c_ *∗*100.

We classified study subjects according to tertiles of ΔGA/HbA_1c_. Individuals in higher tertiles for ΔGA/HbA_1c_ had longer duration of diabetes (2.4 versus 3.7 versus 4.7 years; tertile 1 versus tertile 3, *p* = 0.013) and lower levels of PCGR (3.5 versus 3.5 versus 2.7; tertile 1 versus tertile 3, *p* = 0.011; tertile 2 versus tertile 3, *p* = 0.021) (Figures 2(a) and 2(b)). Moreover, study subjects were categorized into three groups based on duration of diabetes (Group A: ≤6 months, *n* = 153; Group B: >6 months and ≤5 years, *n* = 111; Group C: >5 years, *n* = 76) to investigate the impact of diabetes duration on ΔGA/HbA_1c_ ratio and PCGR. The ΔGA/HbA_1c_ ratios (expressed as percentages) were significantly elevated in patients with diabetes of duration >5 years compared to other groups (Figure 2(c)), whereas PCGR was decreased in patients with longer duration of diabetes (Figure 2(d)).

### 3.4. ΔGA/HbA_1c_ Was Independently Associated with Duration of Diabetes

Multivariable linear regression models were applied to determine the clinical and laboratory variables associated with ΔGA/HbA_1c_ (Table 3). We focused on certain parameters that can directly or indirectly reflect the insulin secretory function, such as PCGR, duration of diabetes, and medication history of DPP-IV inhibitor which can effectively reduce postprandial glucose. After adjustment for clinically important variables such as age, sex, BMI, waist circumference, and estimated GFR in model 1, history of DPP-IV inhibitor use was negatively associated with ΔGA/HbA_1c_ (STD *β* = −0.111, *p* = 0.049). After additional inclusion of PCGR in model 2, PCGR showed significant correlation with ΔGA/HbA_1c_ (STD *β* = −0.161, *p* = 0.009), but history of DPP-IV inhibitor use lost its significance. In model 3, duration of diabetes was further adjusted and the significant correlation of PCGR with ΔGA/HbA_1c_ disappeared (STD *β* = −0.111, *p* = 0.080). However, duration of diabetes was still independently associated with ΔGA/HbA_1c_ (STD *β* = 0.172, *p* = 0.005). Moreover, this association remained significant even after adjustment for glycemic status of subjects (inclusion of mean GA in model 4 and mean HbA_1c_ in model 5, resp.).

Additionally, we conducted multiple linear regression analyses to determine variables associated with PCGR at baseline (Supplementary Table  1 in Supplementary Material available online at http://dx.doi.org/10.1155/2015/576306). PCGR showed the strongest relationship with mean GA (STD *β* = −0.336, *p* < 0.001). It also had significant correlation with duration of diabetes (STD *β* = −0.133, *p* = 0.010) and insulin use (STD *β* = −0.119, *p* = 0.029) (model 1). To evaluate the association between PCGR and ΔGA/HbA_1c_, model 2 was developed, which showed a significant negative relationship (STD *β* = −0.107, *p* = 0.032).

## 4. Discussion

Evidence has accumulated on the clinical relevance of GA as a glycemic index. However, the optimal use of GA as a glucose monitoring tool has not been fully investigated. Based on a previous cross-sectional study that showed that GA values are significantly influenced by the duration of T2D in cases where beta-cell function gradually decreases with time, we hypothesized that the ratio of GA to HbA_1c_ might not be constant over time. In this study of more than 4 years, we assessed glycemic excursion by measuring HbA_1c_ and GA and investigated discrepancy between two glycemic indices according to multiple time points. This study has three main findings: first, we found an initial sharp decrease in these glycemic indices, followed by maintenance at a low level, and then a gradual increase. Unlike for GA, the HbA_1c_ increase was statistically insignificant. Second, the change in GA/HbA_1c_ ratios, defined as the difference between the nadir point and the end point, was independently associated with baseline duration of diabetes. Third, impaired beta-cell function accounted for the association between longer duration of diabetes and increase in GA relative to HbA_1c_, as well as the increase in the GA/HbA_1c_ ratio.

Because HbA_1c_ is formed via a nonenzymatic glycation process of hemoglobin in erythrocytes [12], medical conditions such as pregnancy, hemolytic anemia, chronic kidney disease, or end stage renal disease with dialysis could alter HbA_1c_ levels. In those cases, GA may be a more reliable marker than HbA_1c_ [5]. In contrast to HbA_1c_ formation, which requires intracellular glucose and protein metabolism, GA is formed directly via an extracellular nonenzymatic glycation process in plasma. However, medical conditions associated with albumin metabolism such as obesity, hyperthyroidism, and nephrotic syndrome, as well as glucocorticoid treatment [5], are known to affect GA levels. To avoid complications, we did not include patients with liver cirrhosis, chronic kidney diseases, pregnancy, and hematologic disorders or those who were being treated with steroid therapy.

With respect to the clinical relevance of the GA/HbA_1c_ ratio, it is known that the ratio is significantly correlated with insulin secretory beta-cell function but not with insulin resistance [6]. Recent study also showed that lower insulin secretory capacity predicted increased levels of GA/HbA_1c_ ratio in subjects with T2D [13]. Moreover, the GA/HbA_1c_ ratio in patients with T1D and T2D more accurately reflected glucose excursion [7, 14–16] and diabetic vasculopathy [8, 17] than HbA_1c_ alone. The GA/HbA_1c_ ratio was significantly higher in T2D patients treated with insulin than in those treated with either diet or oral hypoglycemic agents [7, 18]. This observation might explain why history of insulin use is associated with either significant hyperglycemia or decreased beta-cell function. Our study also showed that ΔGA/HbA_1c_ between end point and nadir point is significantly associated with decreased insulin secretory function-related clinical and laboratory variables such as baseline and mean GA, mean HbA_1c_ PCGR, ΔC-peptide, and diabetic duration (Table 2). Of the assessed glycemic indices, baseline HbA_1c_ did not predict the changes in the GA/HbA_1c_ ratio. With respect to the effect of insulin secretory factors on GA values, a recent cross-sectional study reported that GA levels significantly increased more in patients with longer duration of T2D and impaired beta-cell function measured by ΔC-peptide regardless of HbA_1c_ levels [11]. Consistent with this finding, our longitudinal study also showed that patients with higher levels of ΔGA/HbA_1c_ had longer duration of diabetes and lower levels of PCGR (Figure 2). Furthermore, PCGR representing beta-cell function was associated with diabetic duration and insulin use at baseline and mean GA but not with mean HbA_1c_. Based on these findings, we could infer that patients with T2D of longer duration and with higher GA/HbA_1c_ are more likely to have impaired beta-cell function and need insulin.

Our study had several strengths. First, this study is a longitudinal study with a long follow-up period of more than 4 years, which allowed us to investigate the changes in GA and HbA_1c_ levels over time. Second, about 80% of participants had a relatively short duration of diabetes (≤5 years) at enrollment. Lastly, we conducted mixed meal tests to obtain basal and stimulated C-peptide levels, which were then used to calculate PCGR as a measure of beta-cell function. That allowed for standardization of the stimulation calories and glucose content. Because it can be easily calculated and is a reliable indicator of beta-cell function, the PCGR is being used more frequently to help determine the optimal antidiabetic drug treatment [19, 20]. In our study, PCGR levels were strongly associated with ΔC-peptide (*r* = 0.808, *p* < 0.001) which strongly predicted beta-cell function (Supplementary Figure  1). In multivariable linear regression analyses, PCGR was also associated with ΔGA/HbA_1c_. However, because the duration of diabetes strongly affects ΔGA/HbA_1c_, after adjusting for duration of diabetes, the association between ΔGA/HbA_1c_ and PCGR disappeared (Table 3).

This study has the following limitations. First, we did not measure beta-cell function or glucose levels during follow-up period or at the end point. Thus, we did not prove that the difference between GA and HbA_1c_ is caused by a decline in beta-cell function during the follow-up period. Second, since this is a retrospective study, the follow-up period varied among the participants. Third, because we did not assess changes in medication, we could not adjust for its effects.

## 5. Conclusions

We conclude that both impaired beta-cell function and longer duration of diabetes are associated with an increase in GA relative to HbA_1c_ and an increase in the GA/HbA_1c_ ratio. The GA/HbA_1c_ ratio was significantly correlated with insulin secretory beta-cell function and increased as duration of diabetes increased. In this regard, clinicians should be extra careful when interpreting GA and GA/HbA_1c_ ratio values in subjects with longer duration of diabetes. Further well-designed prospective studies enrolling larger populations are warranted.

## Supplementary Material

Supplementary Table 1 Analyses to determine the variables associated with PCGR ^*^Model 1 was adjusted for age (years), sex (0=female, 1=male), body mass index (kg/m2), waist circumference (cm), hypertension (0=no, 1=yes), and estimated glomerular filtration rate (ml/min/1.73m2). ^*^^*^Model 2 was additionally adjusted for ΔGA/HbA1c (end-point, baseline). Supplementary Figure 1 Correlation between ΔC-peptide and PCGR

## Figures and Tables

**Table 1:** Baseline characteristics of the study population.

Variables	All (*N* = 340)
Demographics	
Age (years)	61.3 ± 11.6
Male, *N* (%)	204 (71)
BMI (kg/m^2^)	25.4 ± 3.6
Waist circumference (cm)	88.1 ± 9.0
Hypertension, *N* (%)	195 (57)
Duration of diabetes (years)	1.0 (0–5.0)
Biochemistry profiles	
Creatinine (mg/dL)	0.93 ± 0.2
Estimated GFR (mL/min/1.73 m^2^)	81.5 ± 17.7
Albumin (g/dL)	4.6 ± 0.4
Total cholesterol (mg/dL)	177.2 ± 48.5
Triglyceride (mg/dL)	152.5 ± 110.7
HDL-cholesterol (mg/dL)	47.7 ± 14.3
LDL-cholesterol (mg/dL)	99.7 ± 38.7
Beta-cell function indices at baseline	
Basal glucose (mg/dL)	138.0 ± 50.9
Stimulated glucose (mg/dL)	231.8 ± 87.3
Basal C-peptide (ng/mL)	2.35 ± 1.2
Stimulated C-peptide (ng/mL)	6.50 ± 3.3
ΔC-peptide (ng/ml)	4.13 ± 2.6
PCGR	3.24 ± 2.1
CGI	0.08 ± 0.4
Glycemic indices	
GA at baseline (%)	19.3 ± 6.6
HbA_1c_ at baseline (%)	7.7 ± 1.6
HbA_1c_ at baseline (mmol/mol)	60.8 ± 16.9
GA/HbA_1c_ ratio at baseline	2.47 ± 0.5
GA at end point (%)	16.5 ± 4.9
HbA_1c_ at end point (%)	7.0 ± 1.2
HbA_1c_ at end point (mmol/mol)	53.2 ± 13.1
GA/HbA_1c_ ratio at end point	2.33 ± 0.4
Mean GA (%)	16.5 ± 4.0
Mean HbA_1c_ (%)	7.0 ± 0.9
Medications at baseline	
Insulin, *N* (%)	63 (19)
Metformin, *N* (%)	221 (65)
DPP-IV inhibitor, *N* (%)	59 (17)
Thiazolidinediones, *N* (%)	40 (12)
Sulfonylurea, *N* (%)	88 (26)
Medications at 27 months	
Insulin, *N* (%)	52 (15)
Metformin, *N* (%)	254 (75)
DPP-IV inhibitor, *N* (%)	98 (29)
Thiazolidinediones, *N* (%)	65 (19)
Sulfonylurea, *N* (%)	99 (29)

Continuous variables were described as mean ± SD or median (quartiles), *N* (%) for categorical variables.

BMI, body mass index; GFR, glomerular filtration rate; GA, glycated albumin; CGI, C-peptide-genic index; PCGR, postprandial C-peptide to glucose ratio.

**Table 2:** Univariate linear regression analysis to determine the variables associated with ΔGA/HbA_1c_.

Variables	STD *β*	*p*
Age (year)	0.063	0.246
BMI (kg/m^2^)	−0.063	0.251
Waist circumference (cm)	0.004	0.940
Estimated GFR (mL/min/1.73 m^2^)	−0.032	0.552
Albumin (g/dL)	0.008	0.886
Total cholesterol (mg/dL)	−0.029	0.599
Triglyceride (mg/dL)	−0.080	0.141
HDL-cholesterol (mg/dL)	0.023	0.674
LDL-cholesterol (mg/dL)	−0.007	0.903
GA at baseline (%)	**0.166**	**0.002**
HbA_1c_ at baseline (%)	0.017	0.753
Mean GA (%)	**0.345**	**<0.001**
Mean HbA_1c_ (%)	**0.128**	**0.018**
Duration of diabetes (year)	**0.187**	**0.001**
ΔC-peptide (ng/mL)	−**0.139**	**0.011**
PCGR	−**0.145**	**0.007**
CGI	−0.059	0.284

BMI, body mass index; GFR, glomerular filtration rate; GA, glycated albumin; PCGR, postprandial C-peptide to glucose ratio; CGI, C-peptide-genic index. Values with statistical significance are printed in bold.

**Table 3:** Multivariable linear regression analyses to determine the variables associated with ΔGA/HbA_1c_.

Models	Model 1		Model 2		Model 3		Model 4		Model 5	
Variables	Conventional confounders		Model 1 + PCGR		Model 2 + duration of diabetes		Model 3 + mean GA		Model 3 + mean HbA_1c_	
	STD *β*	*p*	STD *β*	*p*	STD *β*	*p*	STD *β*	*p*	STD *β*	*p*
DPP-IV inhibitor use	**−0.111**	**0.049**	**−**0.109	0.053	**−**0.089	0.111	**−**0.084	0.133	**−**0.088	0.116
PCGR	—	—	**−0.161**	**0.009**	**−**0.111	0.080	**−**0.059	0.396	**−**0.106	0.113
Duration of diabetes	—	—			**0.172**	**0.005**	**0.166**	**0.007**	**0.170**	**0.007**

Conventional confounders: age (years), sex (0 = female, 1 = male), body mass index (kg/m^2^), waist circumference (cm), and estimated glomerular filtration rate (mL/min/1.73 m^2^).

PCGR, postprandial C-peptide to glucose ratio; STD *β*, standardized *β* coefficient. Values with statistical significance are printed in bold.
