# Supplementary material for: Glycated Albumin Levels in Patients with Type 2 Diabetes Increase Relative to HbA1c with Time
Source: Biomed Res Int. 2015 Sep 21;2015:576306. doi: 10.1155/2015/576306 (PMC4592895; doi:10.1155/2015/576306)
Supplement: Supplementary file 1 — Supplementary Table 1 Analyses to determine the variables associated with PCGR *Model 1 was adjusted for age (years), sex (0=female, 1=male), body mass index (kg/m2), waist circumference (cm), hypertension (0=no, 1=yes), and estimated glomerular filtration rate (ml/min/1.73m2). * *Model 2 was additionally adjusted for ΔGA/HbA1c (end-point, baseline). Supplementary Figure 1 Correlation between ΔC-peptide and PCGR [file 576306.f1.pdf]

Supplementary Figure 1 Correlation between ΔC-peptide and PCGR

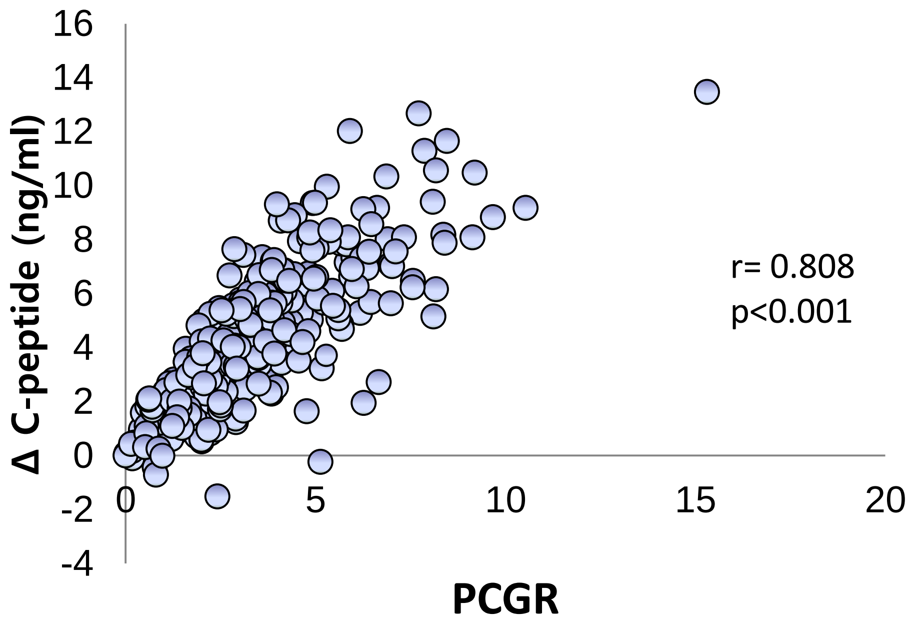

Supplementary Table 1 Analyses to determine the variables associated with PCGR

Variable	Model 1*		Model 2**	
	**STD β**	**P**	**STD β**	**P**
Duration of diabetes (years)	**-0.133**	**0.010**	**-0.212**	**<0.001**
Insulin use at baseline (0=no, 1=yes)	**-0.119**	**0.029**	**-0.264**	**<0.001**
mean GA (%)	**-0.336**	**<0.001**	-	-
mean HbA_{1c} (%)	-0.058	0.452	-	-
ΔGA/HbA_{1c} (end-point, nadir-point)	-	-	**-0.107**	**0.032**

*Model 1 was adjusted for age (years), sex (0=female, 1=male), body mass index (kg/m^2), waist circumference (cm), hypertension (0=no, 1=yes), and estimated glomerular filtration rate (ml/min/1.73m^2). **Model 2 was additionally adjusted for ΔGA/HbA_{1c} (end-point, baseline). Values with statistical significance are printed in bold.
